# Clinical safety and efficacy of neoadjuvant combination chemotherapy of tranilast in advanced esophageal squamous cell carcinoma: Phase I/II study (TNAC)

**DOI:** 10.1097/MD.0000000000023633

**Published:** 2020-12-11

**Authors:** Atsushi Shiozaki, Michihiro Kudou, Hitoshi Fujiwara, Hirotaka Konishi, Hiroki Shimizu, Tomohiro Arita, Toshiyuki Kosuga, Yusuke Yamamoto, Ryo Morimura, Hisashi Ikoma, Yoshiaki Kuriu, Takeshi Kubota, Kazuma Okamoto, Eigo Otsuji

**Affiliations:** Division of Digestive Surgery, Department of Surgery, Kyoto Prefectural University of Medicine, Kyoto, Japan.

**Keywords:** cancer stem cell, esophageal squamous cell carcinoma, preoperative adjuvant chemotherapy, tranilast

## Abstract

**Background:**

Transient receptor potential vanilloid 2 (TRPV2) was previously shown to play an important role in the maintenance of cancer stem cells, and its specific inhibitor, tranilast, also has potential as a targeted therapeutic agent for esophageal squamous cell carcinoma (ESCC). The present study is being conducted to confirm the safety and efficacy of the additional use of tranilast with conventional preoperative adjuvant chemotherapy for patients with advanced ESCC.

**Patients and methods:**

Between 56 and 59 patients aged between 20 and 74 years with clinically diagnosed Stage II or Stage III ESCC will be enrolled. Eligible patients will receive preoperative adjuvant chemotherapy, 2 cycles of combination therapy with cisplatin, 5-fluorouracil, and tranilast. Recruitment started in November 2019, with the final follow-up being planned for March 2029. One subject has been enrolled since October 21^st^, 2020. The pathological therapeutic effect is the primary endpoint. The objective response rate, safety of preoperative adjuvant chemotherapy, recurrence-free survival (RFS), and overall survival (OS) are the secondary endpoints. RFS and OS will be calculated as the time from surgery to first recurrence and all-cause death, respectively.

**Ethics and dissemination:**

This protocol has been approved by the Institutional Review Boards of Kyoto Prefectural University of Medicine and all participating hospitals in August 30, 2019 (Number: CRB5180001). Written informed consent will be obtained from all patients before their registration, which is in accordance with the Declaration of Helsinki. The results of the present study will be disseminated via publication in peer-reviewed journals.

**Trial registration:**

Trial registration number jRCTs051190076

## 1 Introduction

The outcome of esophageal squamous cell carcinoma (ESCC), an aggressive type of neoplasm, has recently been improved in Japan due to advances in multidisciplinary treatments that combine surgery, chemotherapy, and radiotherapy.^[1–3]^ ESCC is a major cause of cancer-related death worldwide, and it frequently recurs in patients with advanced disease, whose prognosis remains poor.^[1–5]^

Despite multidisciplinary treatments, ESCC cells may acquire treatment resistance, persist, and ultimately cause recurrence. Previous studies reported that a large number of solid tumors include a minor subset of cancer cells called cancer stem cells (CSCs).^[6,7]^ These cells have the characteristics of stem cells, such as self-replication ability and pluripotency, exhibit resistance to anticancer drugs and radiation, and are critically involved in tumor initiation, progression, recurrence, and metastasis, ultimately resulting in patient death.^[6,7]^ We previously demonstrated that the expression levels of transient receptor potential vanilloid 2 (TRPV2), a nonspecific cation channel, were very high in the CSCs of ESCC.^[7]^ The cytotoxicity of tranilast, an analog of a tryptophan metabolite and a TRPV2 inhibitor, was shown to be more potent at a lower concentration in CSCs than in non-CSCs.^[7]^ It has been used to treat patients with inflammatory diseases, such as asthma, dermatitis, allergic conjunctivitis, keloids, and hypertrophic scars.^[8]^ The safety of tranilast for clinical use has already been demonstrated.^[9]^

Preoperative adjuvant chemotherapy with cisplatin (CDDP) and 5-fluorouracil (5FU) is currently used with beneficial effects to treat localized advanced ESCC in Japan.^[2,3,10]^ We previously reported that TRPV2 appeared to play a role in maintaining CSCs, and that tranilast, its inhibitor, may enhance the effects of conventional preoperative adjuvant chemotherapy against ESCC.^[7]^ The present study is being performed to confirm the safety and efficacy of the additional use of tranilast with 5-FU/CDDP and to develop a novel therapeutic strategy for patients with advanced ESCC.

## 2 Materials and methods

### 2.1 Study design

The present study is a single arm, open-label prospective intervention phase I/II trial.

### 2.2 Objectives

The purpose of the present study is to confirm the clinical safety and efficacy of the additional use of tranilast with conventional preoperative adjuvant chemotherapy (5-FU/CDDP) for patients with advanced ESCC in a phase I/II design.

### 2.3 Study setting

The study is an investigator-initiated, prospective intervention phase I/II trial to investigate the efficacy of the additional use of tranilast using comparisons with historical control data from patients who previously received conventional preoperative adjuvant chemotherapy (5-FU/CDDP) in our institute. Patients will provide written informed consent before their registration, which is in accordance with the Declaration of Helsinki. Following an independent review by the Center for Quality Assurance in Research and Development, Kyoto Prefectural University of Medicine, patients will be registered in the present study. In accordance with the Japanese clinical trial guidelines, at least annual independent monitoring is planned.

### 2.4 Participants

Patients clinically diagnosed with Stage II or Stage III ESCC and indicated for esophagectomy after preoperative adjuvant chemotherapy will be recruited. The following inclusion criteria are being used:

1. Histologically proven ESCC.
2. Clinically diagnosed Stage II or Stage III ESCC staged according to the 11^th^ edition of the Japanese Classification of Esophageal Cancer.^[11,12]^
3. Patients considered eligible for radical esophagectomy under general anesthesia.
4. Aged between 20 and 74 years at the time of enrollment.
5. Patients with ECOG performance status 0, 1, or 2.
6. Confirmed adequate organ functions within 14 days before enrollment. The following clinical test standards were used:
  a. Creatine ≤1.2 mg/dL
  b. Blood urea nitrogen ≤25 mg/dL
  c. Creatinine clearance ≥60 mL/min
  d. Total bilirubin ≤1.2 mg/dL
  e. Glutamic oxaloacetic transaminase, glutamic pyruvic transaminase ≤twice the upper limit of the institutional reference value
  f. White blood cell count ≥4000 cells/mm^3^
  g. Hemoglobin ≥8.0 g/dL
  h. Platelet count ≥100,000 cells/mm^3^
  i. PaO_2_ ≥70 torr
7. Patients who can take medicine.
8. Oral and written informed consent obtained before registration.

The following exclusion criteria are being applied:

1) History of chemotherapy, radiation, or administration of other investigational agents for synchronous or metachronous malignancies within 1 year before enrollment.
2) Tumor with active bleeding.
3) A patient with a history of acute myocardial infarction, severe angina, congested heart failure, cerebrovascular disease, or pulmonary thrombosis within 6 months before registration.
4) Uncontrollable asthma.
5) History of laparotomy or thoracotomy within 4 weeks before treatment begins.
6) Allergy to tranilast.
7) Females who may be pregnant, breastfeeding an infant, and have a desire to bear children.
8) Any other patients considered to be unsuitable for enrollment by the investigators.
9) A patient who takes some similar drugs to tranilast.
10) A patient who received a transfusion within 14 days before enrollment.
11) Currently being medicated with warfarin.

### 2.5 Dose and treatment regimens of preoperative adjuvant chemotherapy

Preoperative adjuvant chemotherapy will comprise 2 cycles of combination therapy with 80 mg/m^2^ CDDP on day 1, 800 mg/m^2^ 5-FU on days 1 to 5, and 200 to 600 mg/day tranilast on days 1 to 21 every 3 weeks (Table 1).

**Table 1:** Protocol treatment schedule for preoperative adjuvant chemotherapy (1 cycle).

Drug	Dose	Administration method	Administration day
CDDP	80 mg/m^2^	Intravenous drip	Day 1
5-FU	800 mg/m^2^	Intravenous drip	Days 1–5
Tranilast	200–600 mg/body	Oral administration	Days 1-21

5-FU = 5-fluorouracil; CDDP = cisplatin.

Toxicities will be assessed based on the Common Terminology Criteria for Adverse Events (CTCAE) version 5.0. Dose-limiting toxicity (DLT) will be defined by the occurrence of severe toxicities, CTCAE grade 4 or 5. The CTCAE grade 4 adverse reactions which often occur during CDDP and 5FU therapy, such as neutropenia, thrombocytopenia, stomatitis, and diarrhea, will be excluded from DLT.

**Figure 1:**
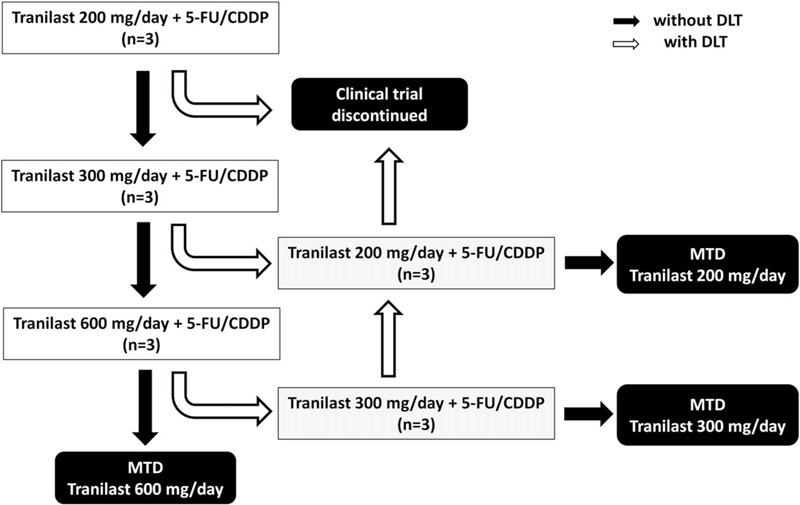
Establishing the maximum tolerated dose (MTD) of tranilast. Initially, 200 mg/day tranilast will be administered to the first 3 patients. If 2 cycles of combination therapy are successfully completed without dose-limiting toxicity (DLT), 300 mg/day tranilast will be administered to the next 3 patients. The dose of tranilast will gradually be increased up to 600 mg/day, and the appropriate MTD will be established. This study will be stopped when DLT occurs at 200 mg/day tranilast. Dose modifications are allowed when DLT occurs at 300 or 600 mg/day tranilast. DLT: grade 4 toxicity according to the Common Terminology Criteria for Adverse Events (CTCAE) version 5.0.

To identify the maximum tolerated dose (MTD) of tranilast, 200 mg/day tranilast will be administered to the first 3 patients. If 2 cycles of combination therapy are successively completed without DLT, 300 mg/day tranilast will be administered to the next 3 patients. The dose of tranilast will gradually be increased to 600 mg/day, and the appropriate MTD will be established (Fig. 1). This clinical study will be stopped when DLT occurs at 200 mg/day tranilast (Fig. 1). Dose modifications will be permitted when DLT occurs at 300 or 600 mg/day tranilast (Fig. 1).

Two cycles of combination therapy with the established MTD of tranilast will then be given to 50 patients. Their data will be compared with historical control data from patients who previously received conventional preoperative adjuvant chemotherapy (5-FU/CDDP) in our institute. This clinical study will be stopped when grade 4 toxicity occurs at the MTD of tranilast in >5 patients.

### 2.6 Rationale for setting the target population size

Between 56 and 59 patients will be enrolled in the present study. An analysis of historical control data from patients who previously received conventional preoperative adjuvant chemotherapy (5-FU/CDDP) in our institute showed good pathological therapeutic effects (tumor regression grade 2 or 3 according to the 11^th^ edition of the Japanese Classification of Esophageal Cancer^[11,12]^) in 19% of patients. Therefore, expected and threshold rates are 34% and 19%, respectively. Under these conditions, 48 subjects will be required based on the assumption of a 1-sided alpha error of 0.05 and beta error of 0.2. In consideration of dropouts, the sample size is set at 50 patients for the phase II trial. Between 9 and 15 patients will be required to establish MTD (phase I trial). Data from 3 of these patients with established MTD will be used for the phase II trial. Therefore, the total sample size has been set at 56 to 59 patients in the present trial.

### 2.7 Data collection methods

Baseline status of physical status of the participants and their baseline findings of their esophageal carcinoma, endoscopy, computed tomography (CT), and positron emission tomography CT (PET-CT), are evaluated before the treatment begins. The patients hospitalize into our hospital just before the start date of the treatment; subsequently, physical status, and laboratory data are regularly checked by the physicians in charge of their hospital care. The status of tumor is re-evaluated using endoscopy, CT, and PET-CT 1 month after the treatment, and chemotherapeutic effect is diagnosed according to RECIST ver 1.1. If unresectable factors are not being found in the reevaluation of tumor, the patients will undergo curative surgery for their esophageal carcinoma. The pathological diagnosis of resected specimens is determined by expert pathologists according to Japanese Classification of Esophageal Cancer, 11th Edition.^[11,12]^ After the surgery, the patients undergo regular follow-up using endoscopy, CT, and PET-CT. The collected data are submitted to data management center by physicians in charge of the hospital care using specific case record forms. The data are managed by the responsible doctor. To protect personal information, the collected information is saved with encrypting the personal data under offline. Oncological outcomes will be investigated at the end of the trial and 3 years later.

### 2.8 Statistical methods

1. ***Population to be analyzed.*** All subjects enrolled in the present study (full analysis set [FAS]), excluding patients with serious violations (including serious protocol deviations, violations of inclusion/exclusion criteria, and violations of prohibited concomitant medication/therapy) from FAS (per protocol set).
2. ***Primary endpoint.*** The primary endpoint is the rate of good pathological therapeutic effect. Primary tumor samples of ESCC will be obtained from patients who have undergone curative esophagectomy after preoperative adjuvant chemotherapy. The grade of the pathological therapeutic effect will be evaluated. Clinical diagnosis according to the 11^th^ edition of the Japanese Classification of Esophageal Cancer^[11,12]^
3. ***Secondary endpoints.*** The secondary endpoints are the objective response rate (ORR), safety of preoperative adjuvant chemotherapy, recurrence-free survival (RFS), and overall survival (OS). RFS and OS will both be calculated as the time from surgery to first recurrence and all-cause death, respectively.
  a. ***ORR.*** Local responses will be evaluated using CT and PET-CT images obtained after preoperative adjuvant chemotherapy in accordance with the 11^th^ edition of the Japanese Classification of Esophageal Cancer.^[11,12]^
  b. ***Safety: Treatment-related adverse event.*** The safety of preoperative adjuvant chemotherapy will be assessed according to CTCAE version 5.0.
  c. ***RFS curve.*** The Kaplan-Meier method will be used to estimate the RFS curve and calculate 3-year RFS and 95% confidence intervals.
  d. ***OS curve.*** The Kaplan-Meier method will be used to estimate the OS curve, and annual and 3-year OS rates and their 95% confidence intervals will be calculated.
4. ***Ethics*** The present trial was approved by the Ethics Committee of Kyoto Prefectural University of Medicine, Kyoto, Japan (Approval number: CRB5180001, 30/Aug/2019). This trial is subject to the supervision and management of the Ethics Committee. If the protocol modifications are needed, the revised protocol must be approved again by CRB. The physicians with sufficient knowledge regarding the present trial obtain informed consent or assent from trial participants using the specific explanation sheet.
5. ***Trial status*** Recruitment started in November 2019, with the final follow-up planned for March 2029. One subject has been enrolled since October 21^st^, 2020.

### 2.9 Quality control and trial monitoring

Before the start of the trial, all researchers will receive special training to guarantee the quality of the study. The training includes how to select and exclude participants, how to implement interventions correctly, how to record case report forms in a standard way, how to assess outcomes and manage data. To improve the compliance of participants, investigators will provide health education, and fully respect the informed consent right of them. Raw data will be recorded in the case record form, and 2 data managers will input the data into the spreadsheet and recheck the data respectively. To ensure the objectivity of the data, the assessment and statistics will be blinded during the trial. The principle researcher will supervise the whole trial.

If adverse events or other unintended effects of the interventions occur, the clinicians in charge need to report the principle researcher immediately, and provide the patient with appropriate therapies for the events. The compensation for participants to adverse event caused by the intervention is provided under sufficient consideration. Monitoring of the trial qualities is performed every 2 months during the trial by the researcher in charge of it.

## 3 Discussion

A recent study demonstrated that ion channels/transporters are critically involved in the various fundamental functions of cancer cells, and are expressed at high levels in CSCs.^[7]^ Our findings on gene expression profiles revealed that the expression levels of several genes encoding ion channels including TRPV2 were elevated in the CSCs of ESCC, suggesting the potential of selective inhibitors of ion channels/transporters as targeted therapeutic agents against CSCs.^[7]^ The inhibitory effects of tranilast on the IgE-induced release of chemical mediators from mast cells have already been demonstrated, and it has been used to treat various inflammatory diseases. Its potential targets include a number of important molecules, such as TRPV2. Tranilast was recently suggested to exhibit antitumor activity against various types of cancers, including breast cancer, gastric cancer, and ESCC.^[7,13]^ Regarding its effects on CSCs, we reported a novel mechanism by which tranilast suppresses CSCs that involves the inhibition of TRPV2.^[7]^

The conventional dose of tranilast for bronchial asthma, allergic rhinitis, atopic dermatitis, keloid, and hypertrophic scars is 300 mg/day (100 mg orally 3 times a day). The internal use of 100 mg tranilast was previously shown to increase the maximal blood level up to 17.1 ± 0.55 μg/mL in healthy subjects. On the other hand, our in vitro study revealed that the 50% inhibitory concentration (IC_50_) was 31.4 μmol/L (10.2 μg/mL), whereas IC_80_ was 113 μmol/L (36.7 μg/mL) in the CSCs of ESCC.^[7]^ If the concentrations of tranilast in the in vitro study correlate with blood levels to some extent, loading for internal use to 2-fold may increase inhibitory effects on CSCs in vivo. Therefore, in the present study, we decided to increase the quantity to double the conventional dose, 600 mg/day, by taking 200 mg orally 3 times a day.

However, increases in the blood level of tranilast have been shown to induce hemorrhagic cystitis because its metabolites cause bladder inflammation. Although caution is needed when increasing the dose of tranilast, a large-scale clinical trial that used 900 mg/day of tranilast as single agent administration has already been conducted.^[10]^ In that clinical trial, the occurrence of side effects was not affected by the loading of tranilast, and, thus, we considered the risk of increments in tranilast in the present study to be within the tolerance level. In consideration of the safety of the additional use of tranilast with 5-FU/CDDP, we decided to start with a low dose of tranilast (200 mg/day), and aim to load up to 600 mg/day. The TNAC study will provide data on appropriate neoadjuvant combination chemotherapy with tranilast for Stage II, III ESCC patients.

## Author contributions

**Conceptualization:** Atsushi Shiozaki, Michihiro Kudou,

**Data curation:** Hitoshi Fujiwara, Hirotaka Konishi, Yusuke Yamamoto, Hisashi Ikoma, Yoshiaki Kuriu, Takeshi Kubota, Kazuma Okamoto

**Investigation:** Atsushi Shiozaki, Michihiro Kudou, Hiroki Shimizu, Tomohiro Arita, Toshiyuki Kosuga

**Methodology:** Atsushi Shiozaki.

**Project administration:** Atsushi Shiozaki, Michihiro Kudou, Hitoshi Fujiwara, Hirotaka Konishi, Hiroki Shimizu, Tomohiro Arita, Toshiyuki Kosuga, Yusuke Yamamoto, Ryo Morimura, Hisashi Ikoma, Yoshiaki Kuriu, Takeshi Kubota, Kazuma Okamoto.

**Supervision:** Eigo Otsuji.

**Validation:** Ryo Morimura, Tomohiro Arita

**Writing – original draft:** Atsushi Shiozaki.

**Writing – review & editing:** Michihiro Kudou, Eigo Otsuji
